# Programmed death ligand 1, poly adenosine diphosphate ribose polymerase, and vascular endothelial growth factor receptor inhibition: a potential combination regimen for targeting triple-negative breast cancer?

**DOI:** 10.1097/JS9.0000000000000550

**Published:** 2023-06-22

**Authors:** Maryam Abdul Wahid, Hassan ul Hussain, Hafsa Ghouri, Tarneem Khan, Syeda Tayyaba Rehan, Hassan Mumtaz

**Affiliations:** a Dow University of Health Sciences; b Karachi Medical and Dental College, Karachi; c Maroof International Hospital. Public Health Scholar, Health Services Academy, Islamabad, Pakistan

## Background

**Figure 1:**
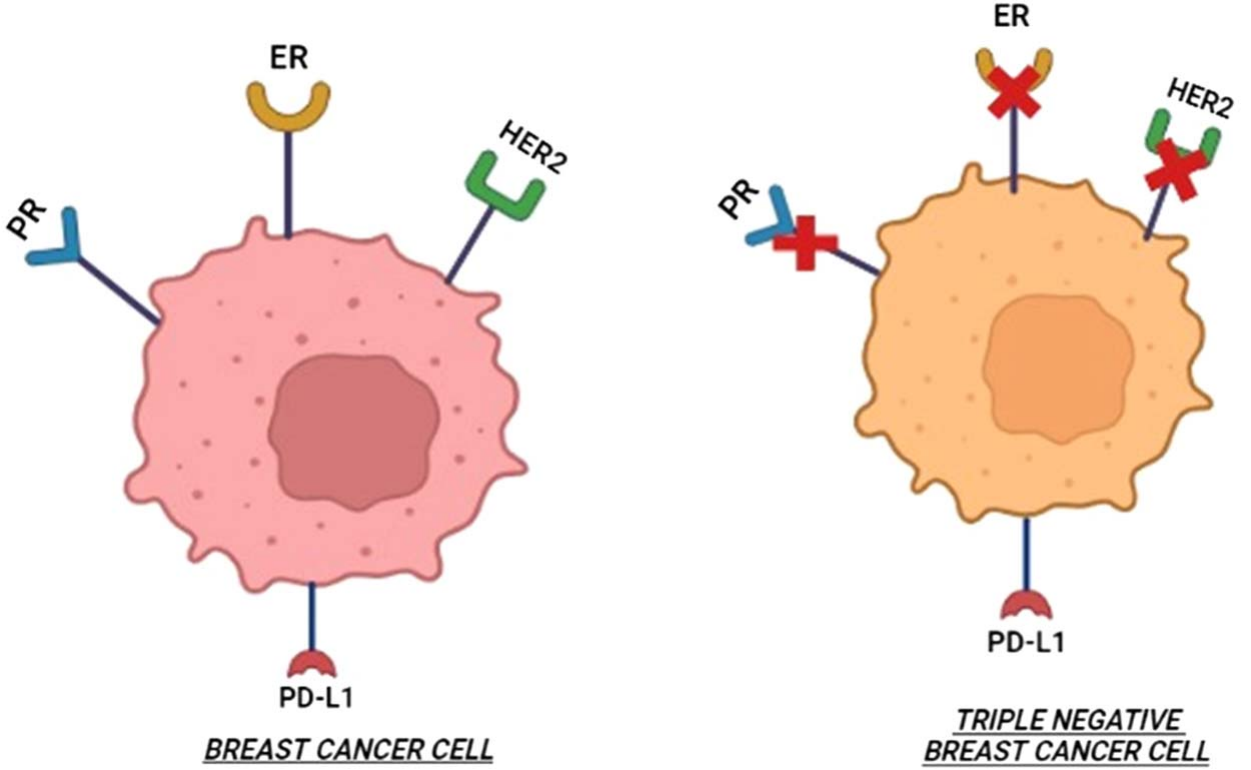
Receptors for triple-negative breast cancer. ER, Estrogen Receptor; HER2, human epidermal growth factor receptor 2; PD-L1, programmed death ligand 1; PR, Progesterone Receptor.

Triple-negative breast cancer (TNBC), an aggressive type of breast cancer (BRCA), has a higher incidence of recurring or metastases. Unlike other BRCAs, tumour cells of TNBC screen negative for oestrogen receptor, progesterone receptor, and human epidermal growth factor receptor 2 as depicted by Figure 1. Therefore, it has a poor prognosis and fewer treatment options. About 10–20% of all BRCAs are TNBC type^1^. It is more prevalent in African American race, mostly in premenopausal young women^2^. It is usually diagnosed at a later stage and associated with high mortality of about 40% population in about 5 years after diagnosis and 75% within 3 months after recurrence^3^. The recurrence rate is 6.7–10.5% within shorter time of 19–40 months as compared with the non-TNBC type which is 35–57 months^4^. Distant metastasis is ~46% in TNBC patients 1–3 years after diagnosis^5^.

## Pathophysiology

Breast cancer gene 1 (BRCA1) is a tumour suppressor gene that plays a role in DNA damage repair and increases the risk of TNBC type of BRCA if mutated. BRCA1 mutations increase Poly (adenosine diphosphate ribose) Polymerase (PARP) that repair DNA damage and promote cancer growth^6^. Molecular characteristic of cancer also bypasses immune responses through various pathways like overexpressing programmed death ligand 1 (PD-L1)^1^. TNBC is also associated with a significant increase in levels of vascular endothelial growth factor (VEGF) resulting in tumour angiogenesis^7^.

## Current therapeutic options

**Figure 2:**
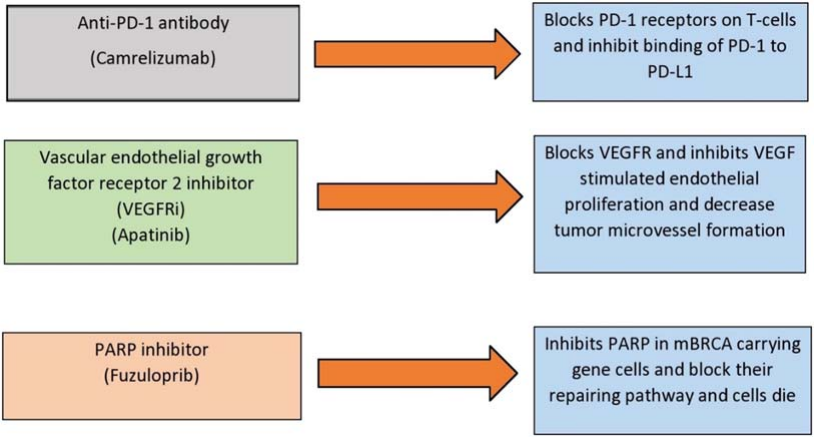
Mechanisms of action of various drugs for treatment of triple-negative breast cancer. mBRCA, BRCA mutation; PARP, poly adenosine diphosphate ribose polymerase; PD-1, programmed death 1; PD-L1, programmed death ligand 1; VEGFRi, vascular endothelial growth factor receptor inhibitor.

TNBC is highly sensitive to chemotherapeutic drugs; hence, chemotherapy remains the standard treatment option, including anthracycline and anthracycline-taxane-based regimens^8^. Another regimen includes a PD-L1 inhibitor, camrelizumab. BRCA1 mutations provide treatment opportunities with the PARP inhibitor, fuzuloparib, which halts DNA break repair. VEGF receptor inhibitor (VEGFRi), apatinib improves tumour responsiveness towards immunotherapy and chemotherapy by inhibiting angiogenesis^8^. Figure 2 displays the mechanisms of action of drug classes used in this combination. Monotherapy with any single drug shows no desirable outcome in metastatic TNBC as compared with combined therapeutic strategies, including PD-L1, VEGFR, and PARP inhibitors, having better safety profiles and promising results^8^.

## Novel combination regimen against TNBC

A recent study investigated the tolerability, safety, preliminary antitumor activity, and pharmacokinetic parameters of camrelizumab plus apatinib and fuzuloparib. It reported that combining a VEGFRi with a PARP inhibitor and an immune checkpoint inhibitor restricted antitumor activity in patients with recurrent or metastatic TNBC^8^.

The study enroled a total of 32 patients with recurrent or metastatic TNBC, with patients in 2 groups; 6 patients in the dose-finding part and 26 in the dose-expansion part (Table 1). The most promising percentage decrease in tumour size from the baseline of the two responders was 79 and 39%^8^.

**Table 1:** Details of patients with recurrent or metastatic TNBC, with patients in two groups; dose finding and dose expansion^8^.

			Due to adverse events				
	Dose details	No. patients	Discontinuation of study treatment	Dose modifications	Mortalities	Dose interruption	Grade ≥ 3 TRAEs
Dose-finding part	Camrelizumab 200 mg + apatinib 375 mg + fuzuloparib 100 mg	3	—	—	—	1	1
Dose expanison part	Camrelizumab 200 mg + apatinib 500 mg + fuzuloparib 100 mg	26	2	4	1	13	17

TNBC, triple-negative breast cancer; TRAEs, Treatment-related adverse event.

The most common treatment-related adverse events reported by different combination therapies were of Grade 3 or higher, including decreased neutrophil count, aspartate aminotransferase and alanine transaminas elevation, fatigue, hand-foot syndrome, and anorexia^8–12^. Further details of treatment-related adverse events and other less common side-effects experienced in various randomized controlled trials (RCTs) are discussed in Table 2.

**Table 2:** Comparing various combinations of drugs targeting angiogenesis in TNBC^8–12^.

			Efficacy				
Combination used	First author	ClinicalTrials.gov registration number	DCR	Median PFS (months)	1-year OSR	ORR	Adverse events
Camrelizumab with apatinib and fuzuloparib	Qingyuan Zhang^8^	NCT03945604	63.30%	5.2	64.20%	2 of 29 patients of 29 had confirmed objective responses (6.9% [95% CI, 0.9–22.8])	Most common TRAEs of grade 3 or higher were decreased WBC count (20.7%), HTN (13.8%), decreased neutrophil count (10.3%), and increased AST (10.3%)
Camrelizumab, apatinib, and eribulin	Jieqiong Liu^10^	NCT04303741	87.00%	8.1	68.30%	37.00%	Grade 3–4 ALT, AST, and bilirubin elevation along with fatigue, rash and hand-foot syndrome
Camrelizumab with apatinib							
Continuous dosing cohort	Jieqiong Liu^9^	NCT03394287	63.30%	3.7	42.20%	43.30%	elevated AST, ALT and hand-foot syndrome. 26.7% and 20.0% of patients experienced grade ≥3 AEs in the continuous dosing and intermittent dosing cohort, respectively
Intermittent dosing cohort			40.00%	1.9	40%	No objective response observed	
Famitinib with camrelizumab and nab-paclitaxel	Li Chen^11^	NCT04129996	95.80%	13.6	82.60%	81.30%	Neutropenia, anaemia, febrile neutropenia, thrombocytopenia, fatigue, and anorexia. Grade 3 peripheral sensory neuropathy was deemed to be taxane-related. Serious TRAEs were with grade 3 septicaemia and grade 3 immune-related myocarditis
Famitinib, camrelizumab and nab-paclitaxel	Song-Yang Wu^12^	NCT04129996		13.6		81.30%	Anorexia, neutropenia, and fatigue, and grade ≥3 AEs

AEs, adverse events; ALT, alanine transaminas; AST, aspartate aminotransferase; DCR, disease control rate; HTN, hypertension; ORR, overall response rate; OSR, overall survival rate; PFS, progression-free survival; TNBC, triple-negative breast cancer; TRAEs, treatment-related adverse events; WBC, white blood cell.

The first trial to report the efficacy and safety of combinational treatment of checkpoint inhibition and antiangiogenesis in TNBC was conducted by Liu *et al.* ^9^ in 2020. It reported the efficacy, safety, and potential predictive biomarkers for programmed death 1 (PD-1) inhibition with camrelizumab, plus apatinib targeting VEGFR2 for patients with advanced TNBC. It is evidenced that camrelizumab with apatinib was well tolerated in advanced TNBC. The results demonstrated a favourable overall response rate and progression-free survival, regardless of lines of therapy and PD-L1 status. Moreover, an overall response rate of 43.3% achieved in the chemo-free regimen was dramatically higher than those in anti-PD-1/PD-L1 antibody (5.2–18.5%) or Apatinib (10.7%) monotherapy^13–15^, demonstrating substantial synergistic effects between these treatments. Details of the efficacy and safety of other clinical trials using various combination regimens are mentioned in Table 2.

## Pharmacokinetics

The pharmacokinetic parameter analysis demonstrated that the absorption of Apatinib was the fastest, and the exposure increased with an increased dosage level from 375 to 500 mg after both single-agent dosing and combined administration with fuzuloparib^8^.

## Usage in other cancers

The combination of angiogenesis inhibition and checkpoint blockade demonstrates increased efficacy, shown through pivotal clinical trials. This combination strategy, approved by the United States Food and Drug Administration (FDA), is now being used in patients with kidney or lung cancer^16,17^. The combination of camrelizumab and apatinib with fuzuloparib can be used in other types of cancers as well after its propitious results in treating platinum-resistant ovarian cancer^18^. The combination therapy of camrelizumab plus apatinib and paclitaxel is also used in cervical cancer^19^.

## Future implications

The current study compares various combinations of drugs for treating recurrent or metastatic TNBC and their effects on other types of cancers. The combination therapy of camrelizumab plus apatinib and fuzuloparib has shown a high efficacy as antitumor activity in TNBC^8^. However, it caused some adverse effects in some patients. Although the incidence of hand-foot syndrome incidence was low in this therapy combination, the sample size of the RCT was small and biomarker analysis was not conducted. Hence, larger RCTs with biomarker analysis are highly recommended to further facilitate the findings of the current RCT.

Hence, this study is highly suggestive of further RCTs and meta-analyses should be conducted on larger sample sizes and biomarker analyses should be performed using all these combination drugs to evaluate long-term efficacy, adverse outcomes, and better treatment plans among patients.

## Ethical approval

Not applicable.

## Consent

Not applicable.

## Source of funding

None.

## Author contribution

M.A.W.: conceptualization; data curation; investigation; visualization; writing—original draft. H.U.H.: data curation; investigation; visualization; review and editing. H.G.: conceptualization; investigation; resources; visualization; supervision. T.K.: data curation; investigation; visualization. S.T.R.: data curation; Writing—original draft. H.M.: supervision; review and editing.

## Conflicts of interest disclosure

None.

## Research registration unique identifying number (UIN)

Not applicable.

## Guarantor

Hassan Mumtaz.

## Data statement

Data will be provided by the authors upon request.

## References

[1] Qi Y Zhang W Jiang R. Efficacy and safety of PD-1 and PD-L1 inhibitors combined with chemotherapy in randomized clinical trials among triple-negative breast cancer. Front Pharmacol 2022;13:960323. 36188589 10.3389/fphar.2022.960323 PMC9523473

[2] Kumar P Aggarwal R. An overview of triple-negative breast cancer. Arch Gynecol Obstetr 2015;293:247–269. 10.1007/s00404-015-3859-y 26341644

[3] Gluz O Liedtke C Gottschalk N. Triple-negative breast cancer—current status and future directions, Ann Oncol 2009;Vol. 20:1913–1927. 10.1093/annonc/mdp492 19901010

[4] Steward L Conant L Gao F. Predictive factors and patterns of recurrence in patients with triple negative breast cancer. Ann Surg Oncol 2014;21:2165–2171. 24558065 10.1245/s10434-014-3546-4

[5] Lin NU Claus E Sohl J. Sites of distant relapse and clinical outcomes in patients with metastatic triple-negative breast cancer: high incidence of central nervous system metastases. Cancer 2008;113:2638. 18833576 10.1002/cncr.23930 PMC2835546

[6] Brouckaert O Wildiers H Floris G. Update on triple-negative breast cancer: prognosis and management strategies. Int J Womens Health 2012;4:511. 23071421 10.2147/IJWH.S18541 PMC3469230

[7] Linderholm BK Hellborg H Johansson U. Significantly higher levels of vascular endothelial growth factor (VEGF) and shorter survival times for patients with primary operable triple-negative breast cancer. Ann Oncol 2009;20:1639–1646. 19549711 10.1093/annonc/mdp062

[8] Zhang Q Shao B Tong Z. A phase Ib study of camrelizumab in combination with apatinib and fuzuloparib in patients with recurrent or metastatic triple-negative breast cancer. BMC Med 2022;20:321. 36184642 10.1186/s12916-022-02527-6 PMC9528096

[9] Liu J Liu Q Li Y. Efficacy and safety of camrelizumab combined with apatinib in advanced triple-negative breast cancer: an open-label phase II trial. J Immunother Cancer 2020;8:e000696. 32448804 10.1136/jitc-2020-000696 PMC7252975

[10] Liu J Wang Y Tian Z. Multicenter phase II trial of Camrelizumab combined with Apatinib and Eribulin in heavily pretreated patients with advanced triple-negative breast cancer. Nat Commun 2022;13:3011. 35641481 10.1038/s41467-022-30569-0 PMC9156739

[11] Chen L Jiang YZ Wu SY. Famitinib with camrelizumab and nab-paclitaxel for advanced immunomodulatory triple-negative breast cancer (FUTURE-C-Plus): an open-label, single-arm, phase II trial. Clin Cancer Res 2022;28:2807–2817. 35247906 10.1158/1078-0432.CCR-21-4313 PMC9365373

[12] Wu SY Xu Y Chen L. Combined angiogenesis and PD-1 inhibition for immunomodulatory TNBC: concept exploration and biomarker analysis in the FUTURE-C-Plus trial. Mol Cancer 2022;21:1–15. 35337339 10.1186/s12943-022-01536-6 PMC8951705

[13] Emens LA Cruz C Paul Eder J. Long-term clinical outcomes and biomarker analyses of atezolizumab therapy for patients with metastatic triple-negative breast cancer: a phase 1 study. JAMA 2019;5:74–82. 10.1001/jamaoncol.2018.4224 PMC6439773 30242306

[14] Dirix LY Takacs I Jerusalem G. Avelumab, an anti-PD-L1 antibody, in patients with locally advanced or metastatic breast cancer: a phase 1b JAVELIN solid tumor study. Breast Cancer Res Treat 2018;167:671–686. 29063313 10.1007/s10549-017-4537-5 PMC5807460

[15] Hu X Zhang J Xu B. Multicenter phase II study of apatinib, a novel VEGFR inhibitor in heavily pretreated patients with metastatic triple‐negative breast cancer. Wiley Online Lib] 2014;135:1961–1969. 10.1002/ijc.28829 24604288

[16] Rini BI Plimack ER Stus V. Pembrolizumab plus axitinib versus sunitinib for advanced renal-cell carcinoma. N Engl J Med 2019;380:1116–1127. 30779529 10.1056/NEJMoa1816714

[17] Socinski MA Jotte RM Cappuzzo F. Atezolizumab for first-line treatment of metastatic nonsquamous NSCLC. N Engl J Med 2018;378:2288–2301. 29863955 10.1056/NEJMoa1716948

[18] Wu Y Zhang X Li L. Case report and review of literature: camrelizumab combined with fuzuloparib and apatinib for platinum-resistant recurrent ovarian cancer. Onco Targets Ther 2022;15:973–979. 36118677 10.2147/OTT.S375643 PMC9480580

[19] Gheorghe AS Dumitrescu EA Komporaly IA. New targeted therapies and combinations of treatments for cervical, endometrial, and ovarian cancers: a year in review. Curr Oncol 2022;29:2835–2847. 35448205 10.3390/curroncol29040231 PMC9027198

